# 

**Published:** 1877-02

**Authors:** 


### EXCERPTA.

---

The firm of Dunlop & Dickson has been dissolved. The undersigned will continue the publication of the Journal as usual. Please address all business communications to

H. H. Dickson.

